# Overexpression of cIAP2 contributes to 5-FU resistance and a poor prognosis in oral squamous cell carcinoma

**DOI:** 10.1038/bjc.2011.387

**Published:** 2011-09-27

**Authors:** M Nagata, H Nakayama, T Tanaka, R Yoshida, Y Yoshitake, D Fukuma, K Kawahara, Y Nakagawa, K Ota, A Hiraki, M Shinohara

**Affiliations:** 1 Department of Oral and Maxillofacial Surgery, Graduate School of Medical Sciences, Kumamoto University, 1-1-1 Honjo, Kumamoto 860-8556, Japan

**Keywords:** cellular inhibitor of apoptosis protein 2, 5-fluorouracil, oral squamous cell carcinoma, resistance, prognosis

## Abstract

**Background:**

Resistance to 5-fluorouracil (5-FU) is a major obstacle in treating oral squamous cell carcinoma (OSCC). However, little is known about apoptosis resistance, which contributes to 5-FU resistance in OSCC.

**Methods:**

We focussed on the cellular inhibitor of apoptosis protein 2 (cIAP2) on the basis of a DNA microarray data using parental and 5-FU-resistant OSCC cell lines. The effects of cIAP2 downregulation on 5-FU sensitivity and apoptosis were evaluated. An immunohistochemical analysis of cIAP2 and related proteins, cIAP1 and X-linked IAP, was performed in 54 OSCC patients who were treated with 5-FU-based chemoradiotherapy and surgery.

**Results:**

The downregulation of cIAP2 significantly enhanced the sensitivity of the 5-FU-resistant cells to 5-FU, with a significant increase in apoptosis. The immunohistochemical analysis demonstrated a high cIAP2 tumour expression to significantly correlate with the pathological response to chemoradiotherapy. Furthermore, a Cox regression analysis revealed the cIAP2 expression status (hazard ratio, 4.91; *P*=0.037) and the pathological response to chemoradiotherapy (hazard ratio, 0.418; *P*=0.016) to be significant prognostic factors for OSCC patients.

**Conclusion:**

These novel findings demonstrate that cIAP2 may represent a potentially useful therapeutic target for improving the treatment and survival of OSCC patients, particularly in the setting of 5-FU resistance.

The widely used chemotherapeutic agent, 5-fluorouracil (5-FU), is important for oral cancer treatment. Clinical studies have shown that 5-FU-based chemotherapy and chemoradiotherapy improve the survival of patients with head and neck cancer, including oral squamous cell carcinoma (OSCC; Pignon *et al*, 2000; Adelstein *et al*, 2006; Tsukuda *et al*, 2010).

However, progressive and recurrent OSCCs show a poor prognosis (Shingaki *et al*, 2003; Bell *et al*, 2007). This is often due to the treatment failure in the setting of progressive, recurrent disease that is resistant to 5-FU-based chemotherapy (Gibson *et al*, 2005; Colevas, 2006). On the other hand, in many cancers that are sensitive to 5-FU, resistance could be ultimately acquired through continuous drug administration (Herrmann, 1996; Kang *et al*, 2004; Yoo *et al*, 2004). In such cases, the drug induces alterations in gene expression and signalling cascades that can mediate resistance (Wang *et al*, 2004; Petersen *et al*, 2010).

One of the hallmark features of cancer is its resistance to apoptosis (Fulda, 2007). However, little is known about apoptosis resistance that contributes to the 5-FU resistance of OSCC. Apoptosis is executed by a family of cysteine proteases known as caspases, which function via two major apoptotic pathways (Chen and Wang, 2002). One is the caspase-8-mediated extrinsic pathway through cell surface death receptors, and the other is the caspase-9-mediated intrinsic pathway. Both pathways converge on the downstream effector caspases: caspases -3, -6, and -7. Chemotherapeutic agents and irradiation are known to induce cell death via the intrinsic pathway (Nachmias *et al*, 2004; Vucic and Fairbrother, 2007).

Inhibitor of apoptosis proteins (IAPs), including cellular IAP1 (cIAP1), cIAP2, X-linked IAP (XIAP), and survivin, are major regulators that block apoptosis by preventing the activation of caspases (Schimmer, 2004). These IAPs directly bind to and inhibit caspases -3, -7, and -9 (Nachmias *et al*, 2004; Schimmer, 2004). Besides acting as direct inhibitors of apoptotic pathways, IAPs have also been implicated in the activation of signal transduction pathways associated with malignancy (Hunter *et al*, 2007; Mahoney *et al*, 2008). The IAPs are positively and negatively regulated by several mechanisms, and they have a differential pattern of gene expression in spite of their structural and functional similarity (Nachmias *et al*, 2004). This phenomenon suggests that the different members of this multigene family have unique functions.

In the present study, to identify novel molecules associated with the 5-FU resistance of OSCC, we established a 5-FU-resistant OSCC cell line over a 2-year period, and found that an overexpression of cIAP2 confers 5-FU resistance in the cells. Furthermore, an immunohistochemical analysis using OSCC patient tissue samples demonstrated that increased expression of cIAP2 resulted in enhanced resistance to 5-FU-based chemoradiotherapy and a poor prognosis.

## Materials and Methods

### Cell line and cell culture

The human OSCC cell line derived from a tongue tumour, SAS, were donated by the Cell Resource Center for Biomedical Research, Tohoku University (Sendai, Japan) and cultured with DMEM supplemented with 10% FBS and maintained under humidified 5% CO_2_ incubation at 37 °C.

### Establishment of 5-FU-resistant OSCC cell lines

To establish 5-FU-resistant cell lines, SAS cells were continuously exposed to increasing concentrations of 5-FU over 2 years. The surviving cells were cloned, and one of the most 5-FU-resistant sublines, designated SAS/FR2, was used for the present studies. The SAS/FR2 can survive exposure to 2.0 *μ*g ml^−1^ 5-FU. To ensure the continued resistance, the cell line was maintained by culture in DMEM containing 2.0 *μ*g ml^−1^ 5-FU. However, to eliminate the effects of 5-FU from the experimental outcomes, the resistant cells were cultured in a drug-free medium for at least 2 weeks before all experiments.

### Cell proliferation assay

To assess their normal proliferation, viable cells without 5-FU treatment were quantified every 24 h using the Cell Counting Kit-8 (Dojindo, Kumamoto, Japan).

### Drug sensitivity assays

The cells (2 × 10^3^ per well) were seeded onto 96-well plates and incubated in DMEM with 10% FBS at 37 °C. After 24 h, DMEM containing various concentrations (0.05, 0.15, 0.31, 0.63, 1.25, 2.5, 5.0, and 10.0*μ*g ml^−1^) of 5-FU was added to each well, then the cells were incubated at 37 °C for another 72 h. For the assay, WST-8 (Cell Counting Kit-8, Dojindo) was added to each well, and the plate was incubated for an additional 2 h at 37 °C. The absorbance was measured at 450 nm using a microplate reader (Model 680, Bio-Rad, Hercules, CA, USA). Eight wells were used for each drug concentration, and the experiment was performed in triplicate. The 50% inhibitory concentration (IC_50_) was calculated from the survival curve.

### Gene expression microarrays

The cRNA was amplified, labelled, and hybridised to a Agilent Human GE 4 × 44K v2 Microarray (Agilent Technologies, Santa Clara, CA, USA) according to the manufacturer's instructions. All hybridised microarrays were scanned by an Agilent scanner and signals of all probes were calculated using Feature Extraction Software (9.5.1.1) (Agilent Technologies).

### Total RNA extraction and reverse transcription PCR

Total RNA was isolated using the RNeasy Mini kit (Qiagen, Valencia, CA, USA). RNA quantity, purity, and integrity were evaluated using a NanoDrop spectrophotometer (Thermo Fisher Scientific, Waltham, MA, USA). Complementary DNA was synthesised from total RNA using the PrimeScript RT reagent kit (Takara Bio Inc., Shiga, Japan). Gene-specific primer sets were designed using the Custom Primers software program (Invitrogen, Carlsbad, CA, USA). The primer sequences and the amplification conditions were shown in Supplementary Table 1. The expression of GAPDH was used as an internal control. Band density was measured using an imaging densitometer.

### Western blotting analysis

The whole-cell proteins were separated by 7.5 or 10% SDS–PAGE, transferred onto nitrocellulose membranes, and probed with antibodies against TS (1 : 200; Chemicon International, Temecula, CA, USA), TP (1 : 50; Abgent, San Diego, CA, USA), cIAP1 (1 : 100; Santa Cruz, Santa Cruz, CA, USA), cIAP2 (1 : 200; Santa Cruz), XIAP (1 : 100; Santa Cruz), and *β*-actin (1 : 5000; Sigma, St Louis, MO, USA). After overnight incubation, the membranes were washed and incubated with horseradish peroxidase-conjugated secondary antibodies (Dako, Glostrup, Denmark). Finally, the membranes were washed and visualised using the ECL Plus detection kit (GE Healthcare, Buckinghamshire, UK).

### Activity assays for caspases -3, -8, and -9, and poly-caspase activities

Cell lines were incubated with 5-FU at a concentration of 2.0 *μ*g ml^−1^ or with culture medium alone. At 48 and 60 h after treatment, the activity levels of caspases -3, -8, and -9 were measured by the APOPCYTO caspase -3, -8, and -9 Colorimetric Assay kits (MBL, Nagoya, Japan), respectively. Absorbance was measured at 405 nm with a microplate reader (Model 680, Bio-Rad). At 60 h after treatment, poly-caspase activation assays were performed using the Poly-Caspases FLICA Apoptosis Detection Kit (Immunochemistry Technologies, Bloomington, MN, USA). Hoechst staining was used to isolate a population of cells.

### The analysis of 5-FU-induced apoptotic cell death

The cells were then fixed with 4% paraformaldehyde at RT for 15 min. After blocking endogenous peroxidase, the cells were permeabilised. For the detection of apoptotic cells with nuclear fragmentation, a terminal deoxynucleotidyl transferase (TdT)-mediated deoxyuridine triphosphate (dUTP) nick end-labelling (TUNEL) method was performed using an *in situ* apoptosis detection kit (Takara Bio Inc.).

### Small interfering RNA transfection

The small interfering RNA (siRNA) for cIAP2 and non-targeting negative control siRNA were obtained from Applied Biosystems (Foster City, CA, USA). The sequences of siRNA used for the study were as follows: cIAP2 siRNA-1: sense, 5′-GAUUCGUUCAGAGUCUAAAtt-3′ and antisense, 5′-UUUAGACUCUGAACGAAUCtg-3′ cIAP2 siRNA-2: sense, 5′-CACUCAUUACUUCCGGGUAtt-3′ and antisense, 5′-UACCCGGAAGUAAUGAGUGtg-3′. Silencer Select Negative Control #1 siRNA (Applied Biosystems) was used as a nonspecific control. SAS/FR2 cells were incubated with antibiotic-free DMEM supplemented with 10% FBS, then transfected with the 10 nmol l^−1^ of cIAP2 siRNA or negative control siRNA with the siPORT NeoFX Transfection Agent (Applied Biosystems). Twenty-four hours after transfection, the cells were treated with 5-FU for each analysis, followed by experiments.

### Clinical characteristics of patients and patient samples

Primary oral cancer tissue samples were obtained from 54 advanced OSCC patients who were treated at Kumamoto University Hospital from October 2003 to January 2009. Signed informed consent was obtained from each patient about the use of surgically resected samples for research purposes. All patients were preoperatively treated with a total of 30 Gy of concurrent chemoradiotherapy with 5-FU before undergoing curative surgery. With regard to the chemoradiotherapy, radiotherapy was administered at a daily dose of 2.0 Gy, five times a week for 15 days, and an oral fluorouracil anticancer agent, S-1, was concurrently administered at a dose of 80, 100, or 120 mg per day according to each patient's body surface area for 14 days from the initiation of radiotherapy. All tumours were staged according to the TNM classification of the UICC (2002), and the degree of differentiation was determined according to the grade classification of the WHO.

### Immunohistochemical staining and the analysis of staining

Tissue samples obtained from biopsy specimens before preoperative chemoradiotherapy were used for the immunohistochemical analyses. Using specimens obtained from surgery, histological responses to chemoradiotherapy were graded according to the criteria of Shimosato *et al* (1971). Paraffin sections fixed in 4% paraformaldehyde were heated in an autoclave at 121 °C for 15 min in a 10 *μ*mol l^−1^ citrate buffer solution at pH 6.0. After quenching the endogenous peroxidase activity, the sections were treated for 2 h at RT with 10% normal goat serum. The sections were incubated overnight at 4 °C with rabbit-polyclonal anti-cIAP1 antibody (1 : 100; Santa Cruz), rabbit-polyclonal anti-cIAP2 antibody (1 : 500; Santa Cruz), and rabbit-polyclonal anti-XIAP antibody (1 : 500; Santa Cruz). After applying the Envision+ System HRP (Dako) for 60 min at RT, immunostaining was visualised with diaminobenzidine. The sections were lightly counterstained with haematoxylin.

Immunoreactivity for cIAP1, cIAP2, and XIAP expression was evaluated by three authors (MN, HN, and MS), who had no knowledge of the patient's clinical status. At least 200 tumour cells were scored per × 40 field. The intensity was classified as 0 (no staining), +1 (weak staining), +2 (distinct staining), or +3 (very strong staining). All sections were scored in a semiquantitative manner according to a previously described method (McCarty *et al*, 1986), which reflects both the intensity and percentage of cells staining at each intensity. The sample was classified as positive when the scoring for cIAP1, cIAP2, and XIAP in a given specimen was ⩾20, because a cutoff of 20 showed the most significant association with survival.

### Statistical analysis

The differences in the mean values between the two groups were statistically analysed using Student's *t*-test, whereas the differences in mean values between multiple groups were analysed using one-way ANOVA with Tukey's test. For analysis of cIAP1, cIAP2, and XIAP expression in the OSCC tissues, the *χ*^2^-test was used to determine the association of cIAP1, cIAP2, and XIAP expression with the clinical and pathological variables. Overall survival (OS) was defined as the time from treatment initiation (chemoradiotherapy) to the date of death from any cause. The Kaplan–Meier method was used to estimate the probability of OS as a function of time, and the statistical differences in the survival of subgroups of patients were compared by using the log-rank test. A multivariate survival analysis was performed using the Cox regression model to study the effects of cIAP1, cIAP2, and XIAP expression on the OS. All *P-*values were based on two-tailed statistical analysis, and *P*-values <0.05 were considered statistically significant (^*^P<0.05 and ^**^P<0.01). All statistical analysis was done using the JMP 9 software program (SAS Institute Inc., Cary, NC, USA).

## Results

### Growth of the 5-FU-resistant cell line and the cytotoxic effects of 5-FU

**Figure 1:**
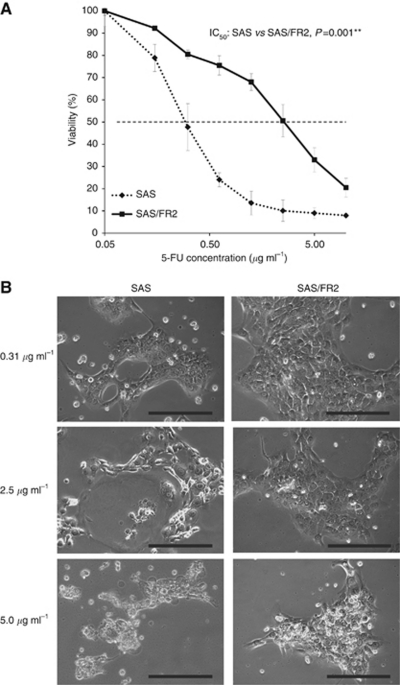
Cytotoxic effects of 5-FU in a 5-FU-resistant cell line, SAS/FR2, and the parent cell line, SAS. (**A**) Cell survival of SAS and SAS/FR2 cells was monitored 72 h after incubation with various concentrations of 5-FU by the Cell Counting Kit-8. The results represent the means±s.d. of three independent experiments. ^**^P<0.01. (**B**) Morphological differences under phase-contrast microscopy between the SAS and SAS/FR2 cells 72 h after 0.31, 2.5, and 5.0 *μ*g ml^–1^ 5-FU treatment. Bar, 100 *μ*m.

The cellular growth activity of the 5-FU-resistant cell line without 5-FU treatment was evaluated for 6 days. No significant difference was found between the cellular growth of the parental (SAS) and resistant (SAS/FR2) cell lines (Supplementary Figure 1), thus suggesting that the 5-FU resistance of OSCC is not due to an increased cell proliferation. We next examined the cytotoxic effects of 5-FU in the SAS and SAS/FR2 cells. Figure 1A shows the drug sensitivity curves in the SAS and SAS/FR2 cells after 72 h of incubation with various concentrations of 5-FU. After 72 h of incubation with 0.31, 2.5, and 5.0 *μ*g l^−1^ 5-FU, increased apoptotic cell changes (shrinkage and rounding up of the cells) were noted in the SAS cells compared with SAS/FR2 cells under phase-contrast microscopy (Figure 1B). The IC_50_ value for 5-FU of the SAS and SAS/FR2 cells was 0.3 and 2.6 *μ*g l^−1^, respectively (*P*<0.01). Therefore, the 5-FU-resistant cell line, SAS/FR2, showed an 8.6-fold higher resistance to 5-FU than the SAS cells.

### The DNA microarray analysis and upregulation of cIAP2

To identify genes differentially expressed between 5-FU-sensitive and -resistant cell lines, a DNA microarray analysis, which contains 40 985 oligonucleotide-based probe sets, was carried out. The results of the analysis showed that the expression levels of 801 genes were elevated and 634 genes were decreased in SAS/FR2 cells, compared with the parental SAS cells. Among these genes, we narrowed our search for the potential targets involved in the function of 5-FU metabolism, drug delivery via the cell membrane, antiapoptotic reactions, and DNA repair. The expression profile of the main target genes involved in those functions identified by DNA microarray analysis are listed in Supplementary Table 2. Thus, we identified three genes (*TS*, 2.6-fold; *TP*, 2.34-fold; and *cIAP2*, 3.78-fold) that were significantly upregulated in SAS/FR2 cells.

As TS and TP are already well known to be involved in 5-FU resistance in many malignancies (Metzger *et al*, 1998; Kawano *et al*, 2003; Longley *et al*, 2003), we focussed on the analysis of cIAP2 in the present study. Because of their structural and functional similarity to cIAP2, cIAP1, and XIAP are especially close IAP family members. Therefore, we then confirmed the expression levels of TS, TP, cIAP1, cIAP2, and XIAP between SAS and SAS/FR2 by a western blotting analysis (Supplementary Figure 2). Consistent with the data obtained from the gene expression analysis, the SAS/FR2 cells clearly expressed higher levels of TS, TP, and cIAP2, with unchanged expression levels of cIAP1 and XIAP compared with the SAS cells.

### An analysis of 5-FU-induced caspases -3, -8, -9, poly-caspase activation, and apoptosis

**Figure 2:**
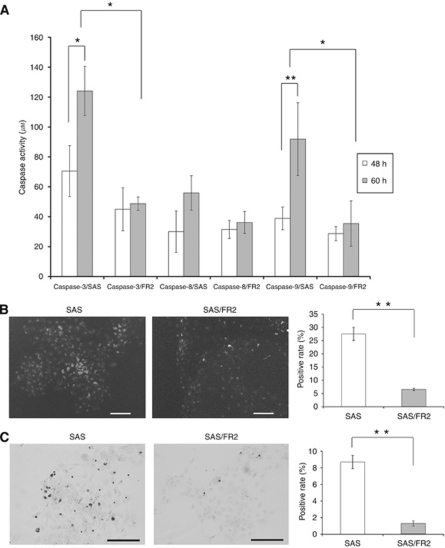
An analysis of 5-FU-induced caspase -3, -8, -9, and poly-caspase activation, and apoptosis in 5-FU-sensitive and -resistant cells. (**A**) Caspase -3, -8, and -9 activity levels were measured using APOPCYTO Colorimetric Assay kits after 48 and 60 h of incubation with 2.0 *μ*g ml^–1^ of 5-FU. (**B**) The activated poly-caspases were visualised by fluorescence microscopy staining after a 60-h incubation with 2.0 *μ*g ml^–1^ of 5-FU. SAS: 27.5% positive and SAS/FR2: 6.5% positive (*P*<0.01). (**C**) *In situ* apoptosis was measured after 72 h with 2.0 *μ*g ml^–1^ 5-FU treatment by TUNEL staining. SAS: 8.7% positive and SAS/FR2: 1.2% positive (*P*<0.01). The results represent the means±s.d. of three independent experiments. ^*^P<0.05 and ^**^P<0.01.

To clarify the differences in 5-FU-induced apoptotic cell death between the cell lines, we analysed the alterations of 5-FU-mediated activation of caspases -3, -8, and -9, and poly-caspase activation (Figure 2A and B). From 48 to 60 h after treatment with 2.0 *μ*g ml^−1^ of 5-FU, the caspase -3 and -9 activities in SAS cells was significantly elevated, whereas the elevation of caspase-8 activity in these cells was not significant. On the other hand, the caspase -3 and -9 activities in the SAS/FR2 cells were elevated to a lesser extent compared with those in the SAS cells. Therefore, at 60 h after 5-FU treatment, the activation levels of caspases -3 and -9 in the SAS/FR2 cells were significantly lower than those in the SAS cells (Figure 2A). Additionally, reflecting the above findings, at 60 h after 5-FU treatment, the SAS/FR2 cells showed significantly decreased positive staining in the poly-caspase activation assay compared with the SAS cells (*P*<0.01, Figure 2B). We next compared apoptotic cell death after 72 h of exposure with 2.0 *μ*g ml^−1^ 5-FU by TUNEL staining (Figure 2C). There were 1.2% and 8.7% TUNEL-positive SAS/FR2 and SAS cells, respectively; therefore the SAS/FR2 cells showed 7.3-fold decreased apoptotic cell death compared with the SAS cells (*P*<0.01).

### Downregulation of cIAP2 increases the chemosensitivity of OSCC to 5-FU

**Figure 3:**
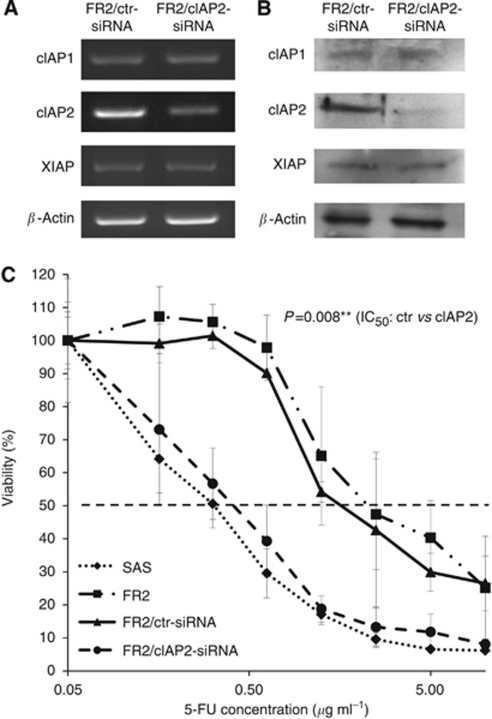
Downregulation of cIAP2 by siRNA increases the chemosensitivity of 5-FU-resistant cells. (**A**) The mRNA levels of cIAP1, cIAP2, and XIAP in SAS/FR2 cells transfected with cIAP2 siRNA or control. At 48 h after transfection, total RNA was extracted, and then the expression levels of each mRNA were analysed by reverse transcription PCR. The relative cIAP2 mRNA level was measured using an imaging densitometer. (**B**) The protein levels of cIAP1, cIAP2, and XIAP in SAS/FR2 cells transfected with cIAP2 siRNA or control. At 72 h after transfection, whole-cell lysates were prepared, and then the expression levels of each protein were examined by a western blotting analysis. (**C**) The enhanced cytotoxic effects of cIAP2 downregulation in 5-FU-resistant cells. At 24 h after transfection with cIAP2 siRNA or control, 5-FU was added at various concentrations. At 72 h after 5-FU treatment, cell survival was monitored. The results represent the means±s.d. of three independent experiments. ^**^P<0.01.

To further elucidate the role of cIAP2 upregulation in 5-FU resistance, we carried out downregulation experiments using siRNA in SAS/FR2 cells. As the data that we obtained from all the experiments using cIAP2 siRNA-1 were similar to those obtained using cIAP2 siRNA-2, we only presented the data for cIAP2 siRNA-1 for the subsequent experiments involving the downregulation of cIAP2. The optimal concentration for the efficient downregulation of cIAP2 was 10 nmol ml^−1^ siRNA, achieving a 70% reduction in mRNA expression, with unchanged expression levels of cIAP1 and XIAP (Figure 3A). Furthermore, we confirmed that there was a significant reduction of cIAP2 protein, without any changes in the expression levels of cIAP1 and XIAP by a western blotting analysis (Figure 3B). On the other hand, there were no differences in either the mRNA or protein expression levels of cIAP2 in SAS/FR2 cells transfected with the control siRNA compared with the untreated cells (data not shown). We then performed a drug sensitivity assay in the cells after transfection with cIAP2 siRNA or negative control siRNA. The cIAP2 targeting significantly enhanced the sensitivity of the cells to 5-FU compared with the control, thus leading to a decrease in the IC_50_ from 1.6 to 0.4 *μ*g ml^−1^ (*P*<0.01, Figure 3C). These results suggest that cIAP2 may be a critical factor affecting the 5-FU resistance of OSCC cells.

### Downregulation of cIAP2 increases 5-FU-induced caspase -3, -9, and poly-caspase activation, and increases apoptosis

**Figure 4:**
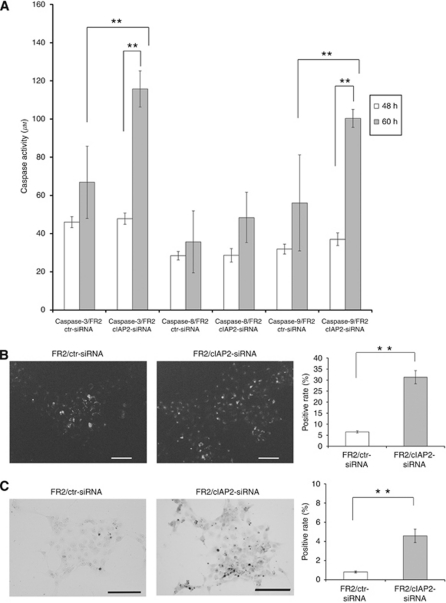
Downregulation of cIAP2 by siRNA increases 5-FU-induced caspase -3, -9, and poly-caspase activation, and increases apoptosis in 5-FU-resistant cells. At 24 h after transfection with the cIAP2 siRNA or control, 2.0 *μ*g ml^–1^ 5-FU was added. (**A**) Caspase -3, -8, and -9 activity levels were measured after incubation with 5-FU for 48 and 60 h. (**B**) The activated poly-caspases were visualised by fluorescence microscopy staining after a 60-h incubation with 5-FU. Control: 6.5% positive and cIAP2 targeting: 31.3% positive (*P*<0.01). (**C**) *In situ* apoptosis was measured after 72 h with 5-FU treatment by TUNEL staining. Control: 0.8% positive and cIAP2 targeting: 4.6% positive (*P*<0.01). The results represent the means±s.d. of three independent experiments. ^**^P<0.01.

To confirm whether the enhanced chemosensitivity to 5-FU by cIAP2 downregulation was due to increased apoptotic cell death, we examined 5-FU-mediated activation of caspases -3, -8, -9, and poly-caspases in the 5-FU-resistant cells after downregulation of cIAP2 via siRNA. From 48 to 60 h after incubation with 2.0 *μ*g ml^−1^ of 5-FU, the caspase -3 and -9 activities in the cIAP2-downregulated cells were significantly elevated compared with the control (Figure 4A). Additionally, reflecting the above results, the cIAP2-downregulated cells showed a significant, 4.9-fold, increase in positive staining compared with the controls in the poly-caspase activation assay (*P*<0.01, Figure 4B). We next confirmed the induction of apoptotic cell death after 72 h of exposure to 2 *μ*g ml^−1^ 5-FU by TUNEL staining (Figure 4C). The percentages of TUNEL-positive cells after transfection with control siRNA and cIAP2 siRNA were 0.8% and 4.6% respectively, indicating that downregulation of cIAP2 induced a 5.8-fold increase in apoptotic cell death (*P*<0.01).

### Clinical significance of cIAP2 expression in the tumours of OSCC patients treated by chemoradiotherapy with 5-FU

**Figure 5:**
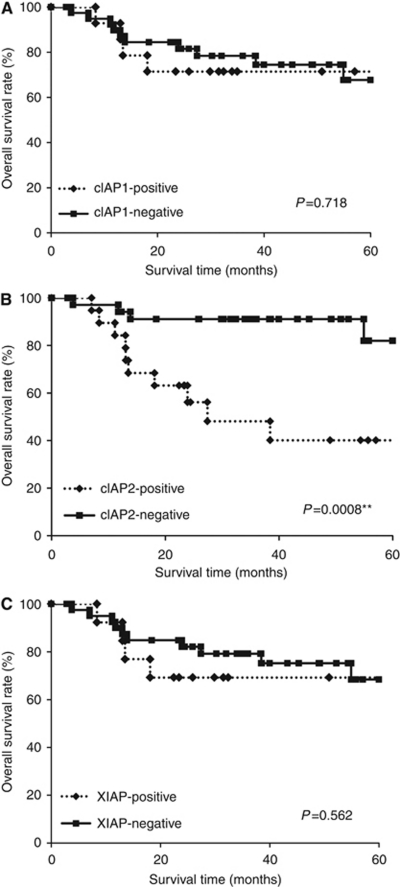
The overall survival of 54 OSCC patients based on their cIAP1 (**A**), cIAP2 (**B**), and XIAP (**C**) status. ^**^P<0.01.

Next, we further examined the expression levels of cIAP1, cIAP2, and XIAP in 54 OSCC patients’ biopsy specimens by immunohistochemical staining. The clinicopathological details of the patients are shown in Table 1. Of the 54 OSCCs we studied, there were 14 (25.9%), 19 (35.1%), and 13 (24.0%) cIAP1-, cIAP2-, and XIAP-positive OSCCs, respectively. The cIAP1, cIAP2, and XIAP expression appeared in the form of a cytoplasmic staining pattern, with some nuclear staining in the tumour cells (Supplementary Figure 3). No distinct tendency towards correlations was observed with any clinicopathological features or with the prognosis in the cases with some nuclear staining (data not shown). The frequency of cIAP2-positive tumours was significantly higher in cases who showed a poor response to preoperative chemoradiotherapy (*P*=0.039), whereas no correlation between the expression status of cIAP1, XIAP, and pathological response to chemoradiotherapy was observed. There were no differences in the expression status of cIAP1, cIAP2, and XIAP according to age, gender, primary tumour site, T stage, clinical stage, or differentiation. However, the overall 5-year survival rate of patients with cIAP2-negative tumours was significantly higher than that of patients with cIAP2-positive tumours (81.9% versus 40.1% *P*=0.0008; Figure 5B), whereas the cIAP1 and XIAP expression status were not associated with the overall 5-year survival rate of OSCC patients (Figure 5A and C). Multivariate analysis using the Cox regression model revealed that the cIAP2 expression status (hazard ratio, 4.91; *P*=0.037) and the pathological response to preoperative chemoradiotherapy (hazard ratio, 0.418; *P*=0.016) were significant prognostic factors for the survival of OSCC patients (Table 2).

## Discussion

To the best of our knowledge, we were the first to establish a 5-FU-resistant OSCC cell line, and the mechanism for 5-FU resistance in OSCC has not been fully elucidated. There are two major methods used to establish 5-FU-resistant cancer cells. One is the pulsatile exposure to high concentrations of 5-FU, and the other is continuous exposure to low concentrations of 5-FU. As a previous report indicated that the pulsatile 5-FU exerts cytotoxicity by an RNA effect, whereas continuous 5-FU is cytotoxic via its effects on DNA synthesis, the mechanisms underlying the 5-FU resistance are believed to differ between these two approaches (Aschele *et al*, 1992). In the present study, 5-FU-resistant cells were developed under continuous exposure to low concentrations of 5-FU to more accurately reflect the clinical setting. We established a 5-FU-resistant OSCC cell line, SAS/FR2, over a 2-year period, and the potency of 5-FU resistance of the cells continued over 3 months after incubation without 5-FU (data not shown). These data indicate that SAS/FR2 is a useful cell line for analysing the biological properties of 5-FU-resistant OSCC.

In the microarray analysis, we narrowed our search to known targets as shown in Supplementary Table 2. Therefore, our data represent only limited data from the comparative gene expression analysis. As a result, we identified three genes, *TS*, *TP*, and *cIAP2*, as 5-FU-resistance-related candidate genes. Both TS and TP were previously demonstrated to be related to 5-FU resistance (Metzger *et al*, 1998; Kawano *et al*, 2003; Longley *et al*, 2003), and are being targeted to increase the treatment efficacy of 5-FU for various cancers. However, since we did not perform experiments to analyse the function of TS or TP in the present study, the effects of these molecules on modulating the 5-FU responsiveness in OSCC are unclear. On the other hand, it is noteworthy that cIAP2 was found to be overexpressed in 5-FU-resistant OSCC, as it may represent a new target that can be used to enhance the sensitivity to 5-FU.

Inhibitor of apoptosis proteins, including cIAP1, cIAP2, XIAP, and survivin, directly inhibit caspases to block apoptosis. Both cIAP1 and cIAP2 are known to be critical regulators of TNF*α*-induced NF-*κ*B activation, which contributes to cell survival (Varfolomeev *et al*, 2008). In addition, the pro-survival effect of NF-*κ*B activation has been linked to the upregulation of several IAPs, including cIAP1, cIAP2, and XIAP (Karin and Lin, 2002). Therefore, it is conceivable that the overexpression of IAPs may be linked to chemoresistance and overall patient prognosis. So far, most of the studies on the importance of IAPs as chemoresponse or prognostic markers has focussed on survivin and XIAP (Sasaki *et al*, 2000; Ferreira *et al*, 2001; Ikeguchi and Kaibara, 2002; Hu *et al*, 2003; Duffy *et al*, 2007; Fulda, 2007; Mizutani *et al*, 2007). On the other hand, there have been some reports that have demonstrated the impact of the upregulation of cIAP1 or cIAP2 on chemoresistance, or decreased patient survival (Krajewska *et al*, 2003; Tanimoto *et al*, 2005). In these studies, chemoresistance has been reported for multiple myeloma (Nakagawa *et al*, 2006), cisplatin resistance in lung cancer (Wu *et al*, 2010), 5-FU resistance in colorectal cancer (Karasawa *et al*, 2009; Miura *et al*, 2009), resistance to cisplatin, doxorubicin, and paclitaxel in pancreatic cancer (Lopes *et al*, 2007), and cisplatin resistance in head and neck cancer (Lee *et al*, 2005). Only Karasawa *et al* have examined the effect of cIAP2 on 5-FU resistance. Furthermore, so far, no other report has demonstrated the contribution of cIAP1 or cIAP2 to both 5-FU resistance and a poor prognosis.

In our *in vitro* data, the 5-FU-resistant cells seemed to exhibit increased expression of cIAP2 as an acquired mechanism of resistance to evade apoptosis under continuous exposure to 5-FU. In fact, 5-FU-induced caspase -3 and -9 activation and -apoptotic cell death were inhibited in 5-FU-resistant OSCC cells which overexpressed cIAP2, and cIAP2 targeting markedly enhanced the cytotoxic activity of 5-FU to the resistant cells through increased caspase -3 and -9 activation. These observations are in agreement with a previous study (Deveraux *et al*, 1998) that showed a role for cIAP2 in apoptosis resistance due to inhibition of the intrinsic apoptotic pathway, and indicated that elevated expression of cIAP2 confers 5-FU resistance in OSCC. To the best of our knowledge, this is the first report to show that increased cIAP2 expression contribute to the resistance to 5-FU in oral cancer.

In previous reports based on comparative analyses between 5-FU-resistant cells and parental cells using DNA microarrays, there was only one report that showed the upregulation of cIAP2, and that study was performed in colorectal cancer cells (Karasawa *et al*, 2009). Taken together, although little is known about the differential functions between cIAP1 and cIAP2, the preferential upregulation of cIAP2 in 5-FU-resistant cancer cells might suggest that it is an important property of the acquired resistance to 5-FU.

However, the major limitation of our study is that the present *in vitro* data are based on the findings restricted to one pair of parental and resistant OSCC cell lines. Therefore, further studies are required to confirm the role of cIAP2 in 5-FU resistance in OSCC by using other 5-FU-resistant OSCC cell lines.

On the other hand, to further identify the special role of cIAP2 in OSCC tissue based on our *in vitro* data, we performed immunohistochemical staining for cIAP2 and the related proteins, cIAP1 and XIAP, using human biopsy specimens. Notably, only the overexpression of cIAP2 in OSCC tissues significantly correlated with a poor response to 5-FU-based chemoradiotherapy and a poor prognosis. Although the reason why cIAP2 overexpression had such an effect is unclear, this result seems to reflect tumour- or cell-type-specific differences in the contribution of individual IAP proteins to apoptosis resistance and to signal transduction pathways associated with malignancy.

In conclusion, we have herein highlighted the potential importance of cIAP2 in 5-FU-resistant OSCC. Our data indicate that cIAP2 could be used to predict the response to 5-FU-based chemoradiotherapy in OSCC patients, and could also represent a novel prognostic factor. Therapies targeting cIAP2 in combination with existing 5-FU-based chemoradiotherapy would enhance the responses to treatments in refractory OSCC patients, and could thereby improve the survival rates.

## Figures and Tables

**Table 1:** Distribution of cIAP1, cIAP2, and XIAP expression in patients with OSCC according to their clinicopathological characteristics

**Characteristics**	**Total**	**cIAP1 positive, *n* (%)**	***P*-value**	**cIAP2 positive, *n* (%)**	***P*-value**	**XIAP positive, *n* (%)**	***P*-value**
	54	14 (25.9)		19 (35.1)		13 (24.0)	
							
*Age, years*							
Median	71	73.8		71		72.3	
Range	51–87	51–85		51–82		51–85	
⩽65	19	3 (15.7)	0.158	8 (42.1)	0.432	3 (15.7)	0.294
>65	35	11 (31.4)		11 (31.4)		10 (28.5)	
							
*Gender*							
Male	31	5 (16.1)	0.051	11 (35.5)	0.957	5 (16.1)	0.112
Female	23	9 (39.1)		8 (34.8)		8 (34.8)	
							
*Primary site*							
Tongue	13	4 (30.7)	0.08	3 (23.1)	0.138	4 (30.7)	0.184
Mandible	10	5 (50.0)		7 (70.0)		4 (40.0)	
Maxilla	12	4 (33.3)		4 (33.3)		4 (33.3)	
Oral floor	9	0 (0)		2 (22.2)		0 (0)	
Buccal mucosa	10	1 (10.0)		3 (30.0)		1 (10.0)	
							
*T stage*							
T1, T2	19	5 (26.3)	0.291	7 (36.8)	0.696	4 (21.0)	0.821
T3	18	3 (16.6)		5 (27.8)		4 (22.2)	
T4	17	6 (35.3)		7 (41.2)		5 (29.5)	
							
*Clinical stage*							
II	4	0 (0.0)	0.622	2 (50.0)	0.769	0 (0)	0.41
III	19	4 (21.0)		7 (36.8)		4 (21.0)	
IV	31	10 (32.2)		10 (32.3)		9 (29.0)	
							
*Differentiation*							
Well	40	9 (22.5)	0.379	14 (35.0)	0.961	10 (25.0)	0.787
Moderate	14	5 (35.7)		5 (35.7)		3 (21.4)	
							
*Pathological response*							
Grades 0, I, IIa	12	2 (16.7)	0.276	7 (58.3)	0.039^*^	2 (16.6)	0.173
Grade IIb	17	3 (17.6)		8 (47.1)		2 (11.8)	
Grade III	8	5 (62.5)		2 (25.0)		4 (50.0)	
Grade IV	17	4 (23.5)		2 (11.8)		5 (29.4)	

Abbreviations: cIAP=cellular inhibitor of apoptosis protein; OSCC=oral squamous cell carcinoma; XIAP=X-linked IAP.

^*^P<0.05.

**Table 2:** The results of a multivariate regression analysis for predicting the overall survival of 54 OSCC patients

**Variables**	**Assigned score**	**Hazard ratio**	**95% CI**	***P*-value**
*Age, years*				
<65	0	1.694	0.33–9.87	0.524
⩾65	1			
				
*Gender*				
Male	0	1.704	0.33–10.4	0.523
Female	1			
				
*T stage*				
T1, T2	1	0.962	0.38–2.72	0.936
T3	2			
T4	3			
				
*Clinical stage*				
II, III	1	4.15	0.81–26.4	0.086
IV	2			
				
*Differentiation*				
Well	1	2.134	0.379–10.3	0.37
Moderate	2			
				
*Pathological response*				
Grades 0, I, IIa	1	0.418	0.15–0.86	0.016^*^
Grade IIb	2			
Grade III	3			
Grade IV	4			
				
*cIAP1 status*				
Negative	0	0.42	0.05–2.26	0.329
Positive	1			
				
*cIAP2 status*				
Negative	0	4.91	1.09–29.9	0.037^*^
Positive	1			
				
*XIAP status*				
Negative	0	0.324	0.37–16.7	0.324
Positive	1			

Abbreviations: CI=confidence interval; cIAP=cellular inhibitor of apoptosis protein; OSCC=oral squamous cell carcinoma; XIAP=X-linked IAP. ^*^P<0.05.
