# Supplementary figures and images for: Overexpression of cIAP2 contributes to 5-FU resistance and a poor prognosis in oral squamous cell carcinoma
Source: Br J Cancer. 2011 Sep 27;105(9):1322–30. doi: 10.1038/bjc.2011.387 (PMC3241556; doi:10.1038/bjc.2011.387)

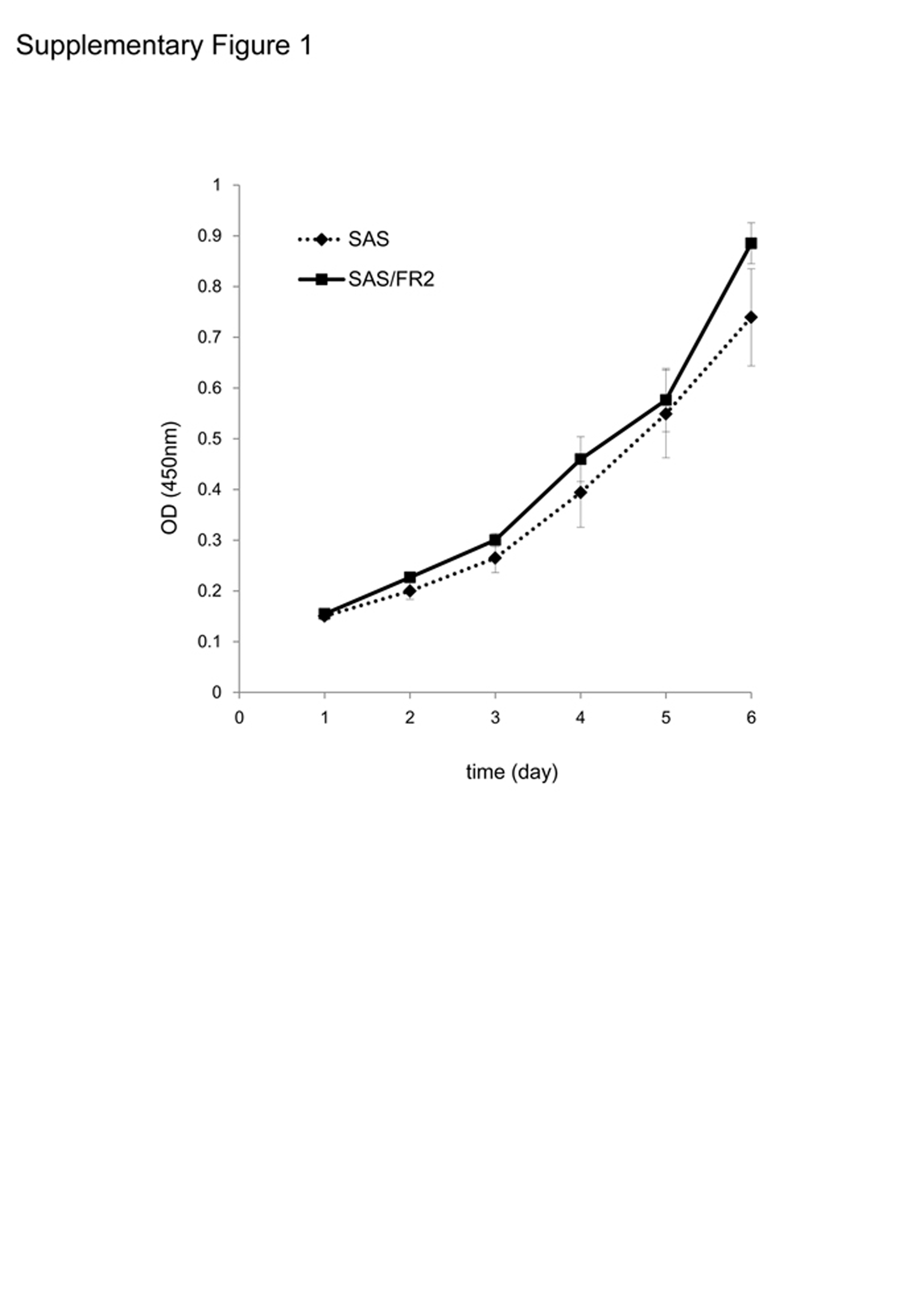

Supplement: Supplementary Figure 1 [file bjc2011387x1.tif]

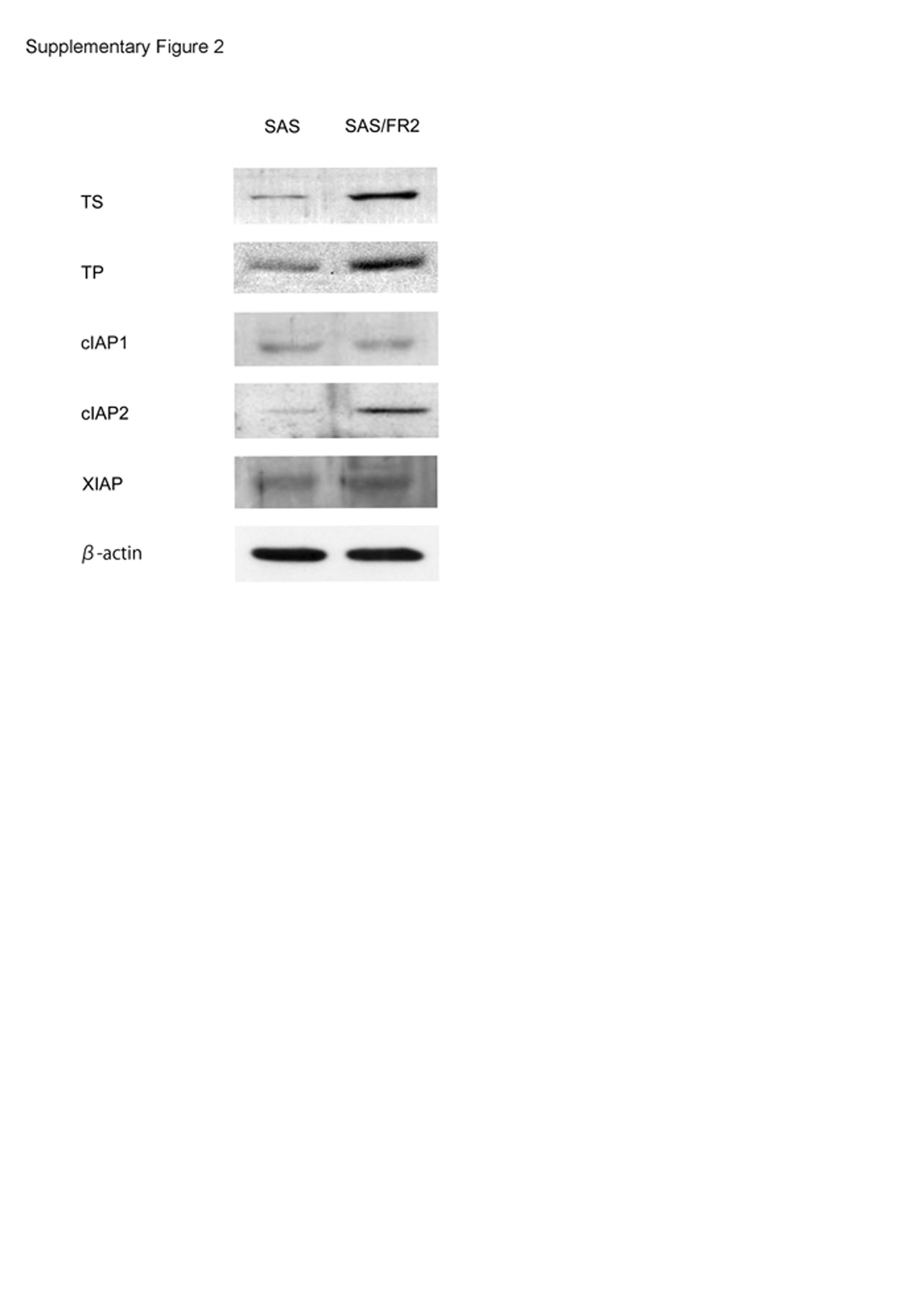

Supplement: Supplementary Figure 2 [file bjc2011387x2.tif]

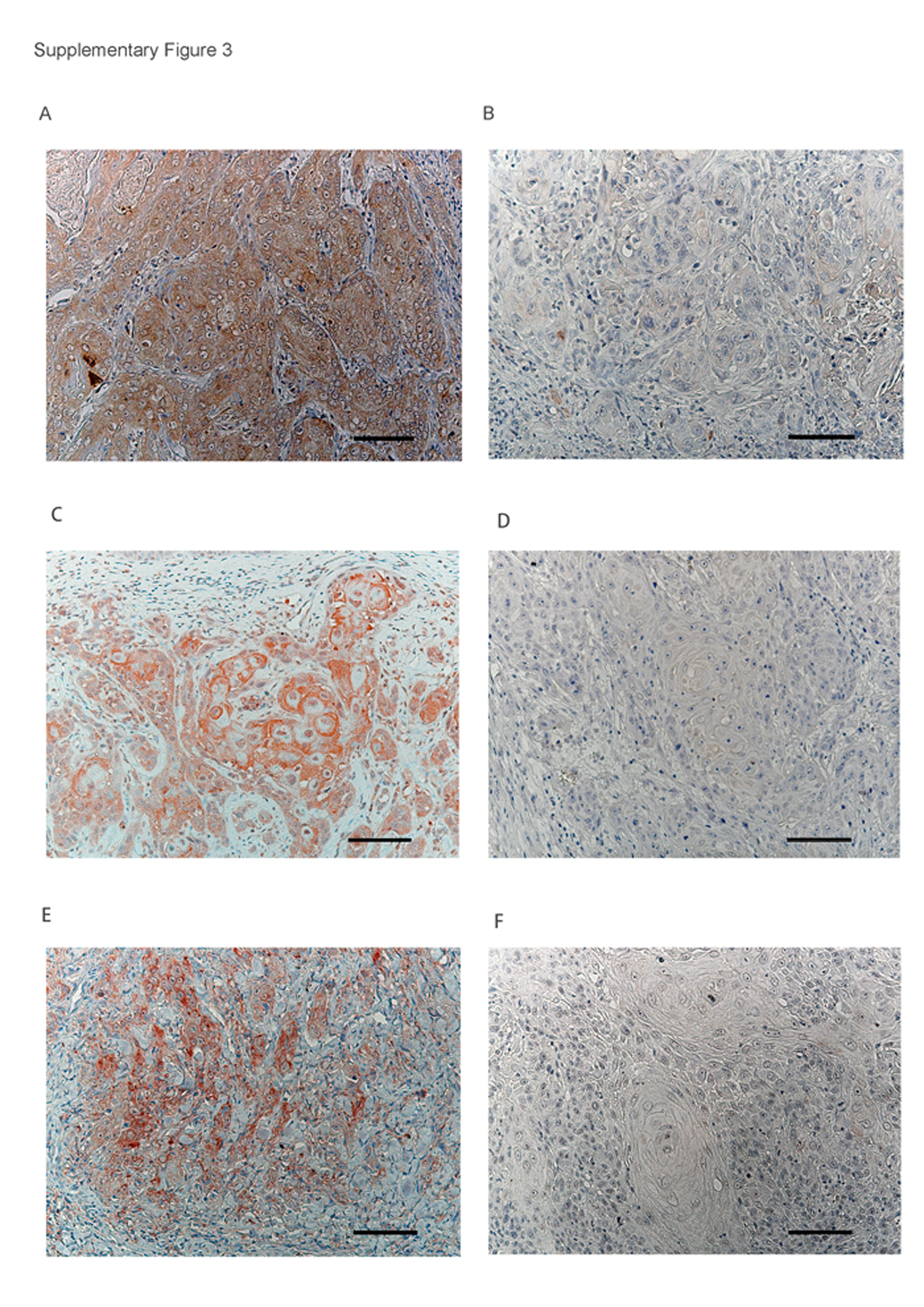

Supplement: Supplementary Figure 3 [file bjc2011387x3.tif]

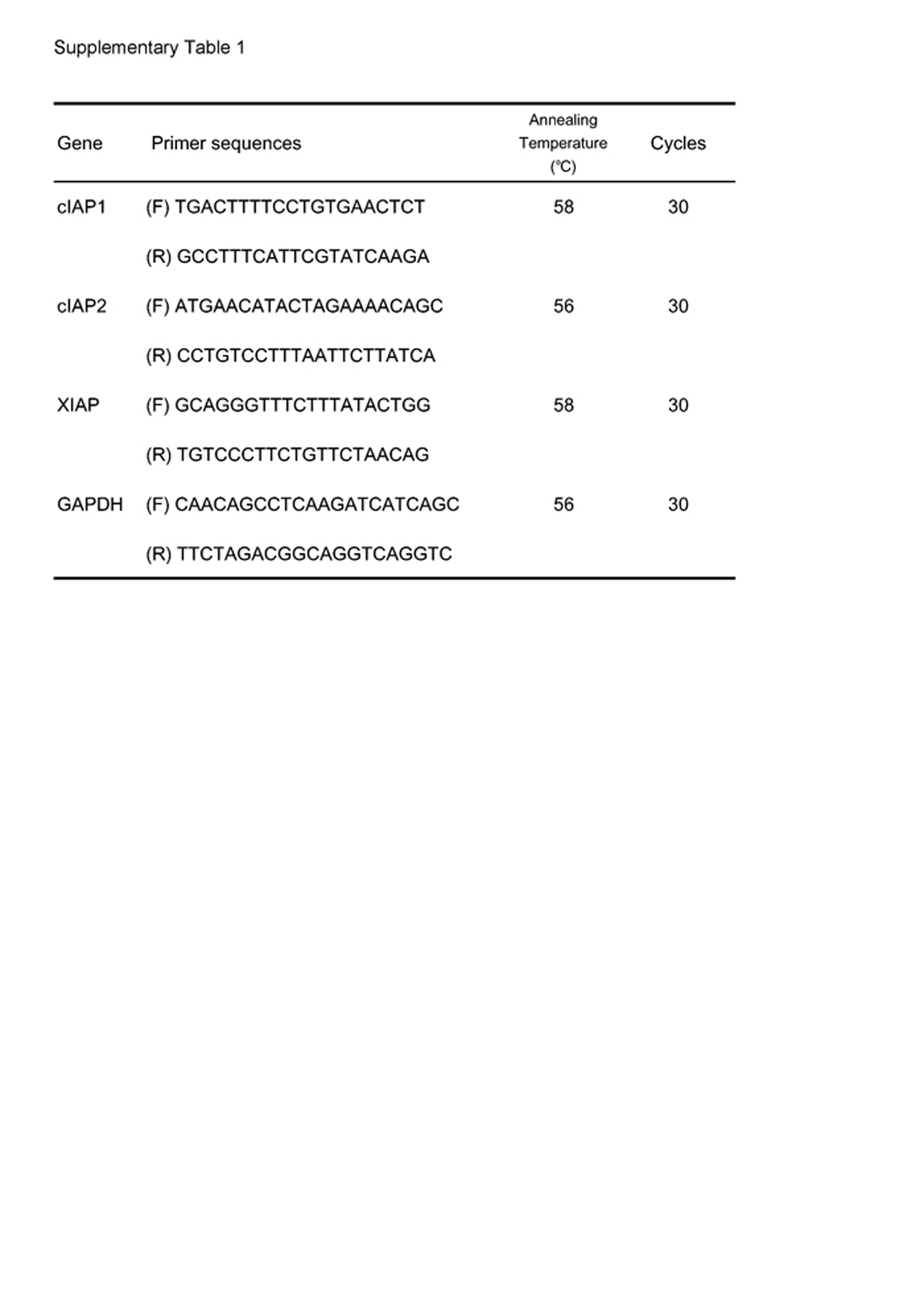

Supplement: Supplementary Table 1 [file bjc2011387x4.tif]

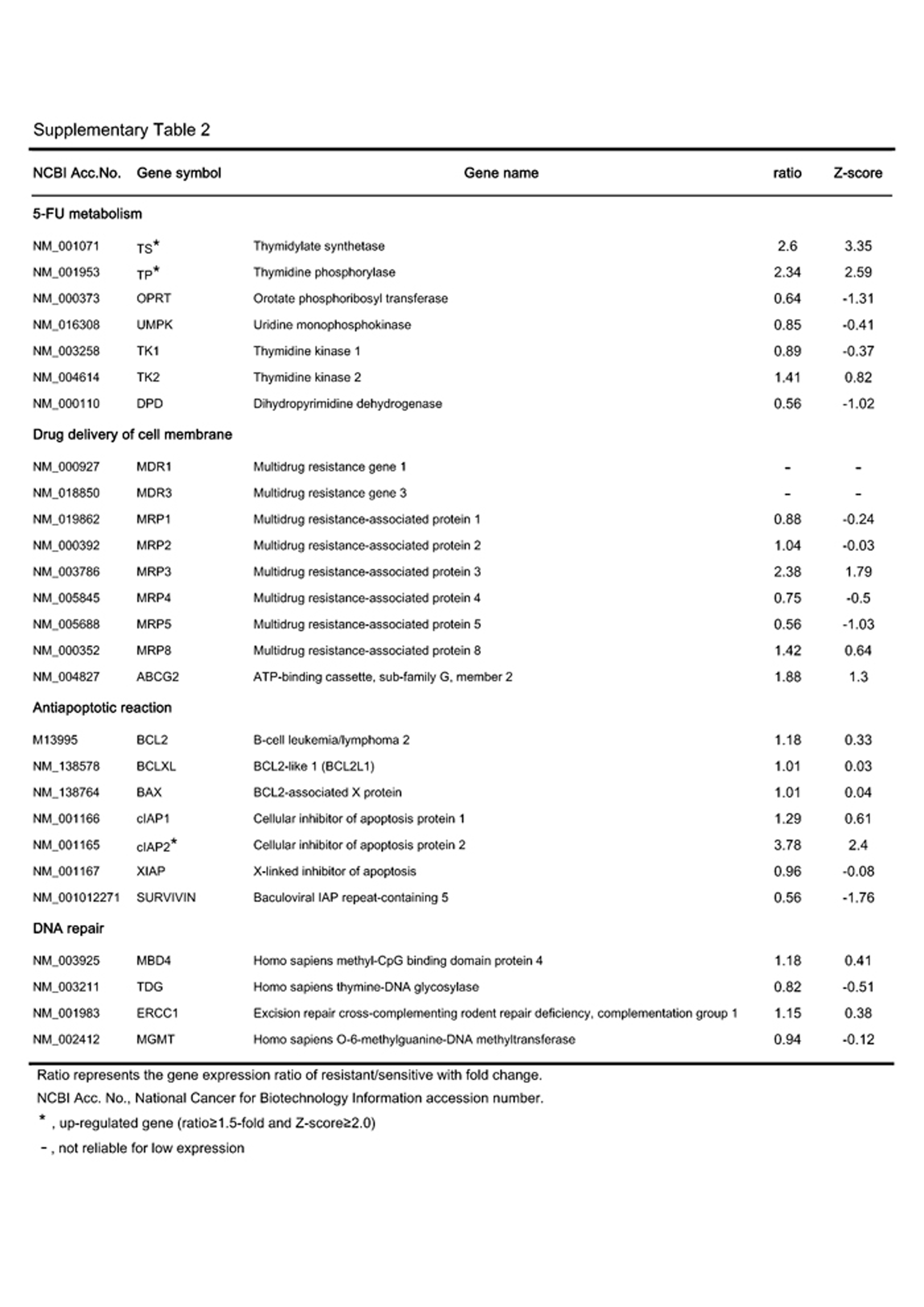

Supplement: Supplementary Table 2 [file bjc2011387x5.tif]
