# Supplementary material for: Overexpression of cIAP2 contributes to 5-FU resistance and a poor prognosis in oral squamous cell carcinoma
Source: Br J Cancer. 2011 Sep 27;105(9):1322–30. doi: 10.1038/bjc.2011.387 (PMC3241556; doi:10.1038/bjc.2011.387)
Supplement: Supplementary Information [file bjc2011387x6.doc]

**Supplementary materials Materials and methods**

**Data analysis and filter criteria**

Raw signal intensities and flags for each probe were calculated from the hybridization intensities, and spot information, according to the procedures recommended by Agilent. And the raw signal intensities of two samples were log2-transformed and normalized by a quantile algorithm (Bolst*ad et* al, 2003) on the Bioconductor (Gentlem*an et* al, 2004; Quackenbush, 2002). We selected probes that called the ‘P’ flag in both control and experimental samples. To identify up or down-regulated genes, we calculated Z-scores (Quackenbush, 2002) and ratios (non-log scaled fold-change) from the normalized signal intensities of each probe. We thereafter established the criteria for the regulated genes: (up-regulated genes) Z-score ≥ 2.0 and ratio ≥ 1.5-fold, (down-regulated genes) Z-score ≤ -2.0 and ratio ≤ 0.66.

Supplementary Table 1

|  |  |  |  |
| --- | --- | --- | --- |
| Gene | Primer sequences | Annealing Temperature (℃) | Cycles |
| cIAP1 | (F) TGACTTTTCCTGTGAACTCT | 58 | 30 |
|  | (R) GCCTTTCATTCGTATCAAGA |  |  |
| cIAP2 | (F) ATGAACATACTAGAAAACAGC | 56 | 30 |
|  | (R) CCTGTCCTTTAATTCTTATCA |  |  |
| XIAP | (F) GCAGGGTTTCTTTATACTGG | 58 | 30 |
|  | (R) TGTCCCTTCTGTTCTAACAG |  |  |
| GAPDH | (F) CAACAGCCTCAAGATCATCAGC | 56 | 30 |
|  | (R) TTCTAGACGGCAGGTCAGGTC |  |  |
|  |  |  |  |

Supplementary Table 2

| **NCBI Acc.No.** | **Gene symbol** | **Gene name** | **ratio** | **Z-score** |
| --- | --- | --- | --- | --- |
| **5-FU metabolism** | |  |  |  |
| NM_001071 | TS* | Thymidylate synthetase | 2.6 | 3.35 |
| NM_001953 | TP* | Thymidine phosphorylase | 2.34 | 2.59 |
| NM_000373 | OPRT | Orotate phosphoribosyl transferase | 0.64 | -1.31 |
| NM_016308 | UMPK | Uridine monophosphokinase | 0.85 | -0.41 |
| NM_003258 | TK1 | Thymidine kinase 1 | 0.89 | -0.37 |
| NM_004614 | TK2 | Thymidine kinase 2 | 1.41 | 0.82 |
| NM_000110 | DPD | Dihydropyrimidine dehydrogenase | 0.56 | -1.02 |
| **Drug delivery of cell membrane** | | |  |  |
| NM_000927 | MDR1 | Multidrug resistance gene 1 | - | - |
| NM_018850 | MDR3 | Multidrug resistance gene 3 | - | - |
| NM_019862 | MRP1 | Multidrug resistance-associated protein 1 | 0.88 | -0.24 |
| NM_000392 | MRP2 | Multidrug resistance-associated protein 2 | 1.04 | -0.03 |
| NM_003786 | MRP3 | Multidrug resistance-associated protein 3 | 2.38 | 1.79 |
| NM_005845 | MRP4 | Multidrug resistance-associated protein 4 | 0.75 | -0.5 |
| NM_005688 | MRP5 | Multidrug resistance-associated protein 5 | 0.56 | -1.03 |
| NM_000352 | MRP8 | Multidrug resistance-associated protein 8 | 1.42 | 0.64 |
| NM_004827 | ABCG2 | ATP-binding cassette, sub-family G, member 2 | 1.88 | 1.3 |
| **Antiapoptotic reaction** | |  |  |  |
| M13995 | BCL2 | B-cell leukemia/lymphoma 2 | 1.18 | 0.33 |
| NM_138578 | BCLXL | BCL2-like 1 (BCL2L1) | 1.01 | 0.03 |
| NM_138764 | BAX | BCL2-associated X protein | 1.01 | 0.04 |
| NM_001166 | cIAP1 | Cellular inhibitor of apoptosis protein 1 | 1.29 | 0.61 |
| NM_001165 | cIAP2* | Cellular inhibitor of apoptosis protein 2 | 3.78 | 2.4 |
| NM_001167 | XIAP | X-linked inhibitor of apoptosis | 0.96 | -0.08 |
| NM_001012271 | SURVIVIN | Baculoviral IAP repeat-containing 5 | 0.56 | -1.76 |
| **DNA repair** |  |  |  |  |
| NM_003925 | MBD4 | Homo sapiens methyl-CpG binding domain protein 4 | 1.18 | 0.41 |
| NM_003211 | TDG | Homo sapiens thymine-DNA glycosylase | 0.82 | -0.51 |
| NM_001983 | ERCC1 | Excision repair cross-complementing rodent repair deficiency, complementation group 1 | 1.15 | 0.38 |
| NM_002412 | MGMT | Homo sapiens O-6-methylguanine-DNA methyltransferase | 0.94 | -0.12 |
| Ratio represents the gene expression ratio of resistant/sensitive with fold change. | | | | |
| NCBI Acc. No., National Cancer for Biotechnology Information accession number. | | | | |
| ＊, up-regulated gene (ratio≥1.5-fold and Z-score≥2.0) | | |  |  |
| - , not reliable for low expression | | |  |  |

**Supplementary Table titles**

**Supplementary Table 1. The primer sequences and amplification conditions for RT-PCR**

**Supplementary Table 2. Comparison of 5-FU-resistance-related genes between 5-FU-sensitive and -resistant cell lines by a cDNA microarray analysis.**

**Supplementary Figure legends**

**Supplementary Figure 1. The cellular growth activities of the parental and 5-FU-resistant cell lines without 5-FU treatment.** Cell proliferation was monitored for 6 days by the Cell Counting Kit-8. The results represent the means ± SD of three independent experiments.

**Supplementary Figure 2. A comparison of TS, TP, cIAP1, cIAP2, and XIAP proteins between 5-FU-sensitive and -resistant cell lines by a Western blotting analysis.** The expression of β-actin was used as an internal loading control.

**Supplementary Figure 3. Immunohistochemical staining of cIAP1, cIAP2, and XIAP in human OSCC tissues.** Representative images of cancer cells with positive and negative expression of cIAP1 (A, B), cIAP2 (C, D), and XIAP (E, F). Bar, 100 μm.
